# Effect of the yellow passion fruit peel flour (*Passiflora edulis* f*. flavicarpa* deg*.*) in insulin sensitivity in type 2 diabetes mellitus patients

**DOI:** 10.1186/1475-2891-11-89

**Published:** 2012-10-22

**Authors:** Maria do Socoro Ramos de Queiroz, Daniele Idalino Janebro, Maria Auxiliadora Lins da Cunha, Josimar dos Santos Medeiros, Armando UO Sabaa-Srur, Margareth de Fatima FM Diniz, Silvana Cristina dos Santos

**Affiliations:** 1 Department of Pharmacy, Paraíba State University, Zip Code: 58429-600, Campina Grande, PB, Brazil; 2 Maurício de Nassau College, Zip Code 58410-050, Campina Grande, PB, Brazil; 3 Department of Nutrition, Federal University of Rio de Janeiro, Zip Code 21949-900, Rio de Janeiro, PB, Brazil; 4 Department of Pharmacy, Paraíba State University, Zip Code: 58051-970, Campina Grande, PB, Brazil

## Abstract

**Background:**

A study with the yellow passion fruit peel flour showed positive action in blood glucose control as therapies’ adjuvant in patients with type 2 diabetes mellitus. Therefore, we evaluated its effect on insulin sensitivity since there is a quest for studies that focus at better understanding of insulin resistance aspects in diabetic patients. Furthermore its relationship with chronic complications can also give good prospects for alternative treatments.

**Methods:**

A total of 43 type 2 diabetes volunteers (28 females and 15 males) ingested 30 g/day of the yellow passion fruit peel flour for two months. The levels of blood glucose and fasting insulin, HOMA index and glycated hemoglobin were measured for each patient before and after dietary supplementation.

**Results:**

There was a significant difference in the fasting blood glucose values (P = 0.000) and glycated hemoglobin (P = 0.032) after supplementation. It was also seen a reduction in HOMA IR (P = 0.005) in the supplemented group, however it was not observed changes in insulin values for females. HOMA beta (P = 0.000) showed significant increase in its values for the studied group.

**Conclusions:**

The supplementation used decreased insulin resistance in type 2 diabetic patients, suggesting a positive action in blood glucose control as adjuvant therapy in conventional treatments.

## Background

Epidemiological studies have shown that fiber rich diets are associated with a reduced risk of diabetes and cardiovascular disease (CVD) [1-8] and it is inversely related to insulin resistance (IR) [9]. However, the metabolic pathway to enlighten the effects of dietary fiber remains without a noteworthy explanation [10].

Pectin is a soluble fiber widely used as an ingredient in pharmaceutical preparations as antidiarrheal and detoxifying substance. Furthermore, it reduces glucose intolerance in diabetic patients and decreases serum cholesterol and triglycerides levels [11,12] by forming a gel which prevents the absorption of cholesterol and glucose derived from the diet [10].

The passion fruit (*Passiflora edulis* f. *flavicarpa* Deg.) peel flour is rich in soluble fiber and has pectin as one of its components [13]. Normal e diabetic guinea pigs fed with the flour presented diabetes control, due to its hypoglycemic action [14]. A clinical toxicological assay of this flour was also performed with a daily intake of 30 g and showed no acute or subchronic toxicity, suggesting its use as a dietary supplement, making it feasible to carry out further efficacy studies in people with diabetes because it is a product rich in soluble fiber [15].

Considering the yellow passion fruit peel flour, in some studies, showed hypoglycemic action and can be used as a dietary supplement, this study investigated its effect on insulin sensitivity since the pursuit for research projects aimed at better understanding IR aspects in diabetic patients and its relationship with chronic complications can also give good perspectives for alternative treatments.

## Methods

### Clinical data

The study was approved by the Paraiba State University Ethics Committee (Opinion No. 0146.0.133.000-07). It was a phase II and cross-interventionist clinical trial, using the flour of the yellow passion fruit peel as a food supplement. The flour was produced by “A.S.S. Neto’s Alimentos Ltda” (CNPJ 82.112.708/0001-20) located in Rio de Janeiro under the guidance of Professor Dr. Armando Oliveira Ubirajara Sabaa Srur.

The patients were classified according to the American Diabetic Association (ADA) [16] and their selection occurred through random sampling from the population attended by the Pharmaceutical Care Program (PROATENFAR) from the Paraiba State University (UEPB). The study excluded patients considered unfit during the interview and/or physical examination, which showed changes in laboratory tests such as: liver and renal dysfunction, cardiac severe changes, alcoholism and those who changed diet, medical treatment or physical activity during the study.

From June 2007 to June 2008, 60 patients were evaluated, 36 women and 24 men, aged between 57 and 73 years, irrespective of ethnicity. Of these, 43 volunteers remained at the end of the experiment, 28 female and 15 male. People who did not remain until the end abandoned the study due to the strong aftertaste of the flour and abdominal discomfort. This has also been reported in other studies and it is probably due to the hesperidin, a substance present in the passion fruit mesocarp, believed to be responsible for the residual bitter taste in the flour [17]. Future trials need to be developed in order to improve the taste, thereby contributing to a better acceptance of the flour and hence the formulation of functional foods.

Throughout the study, 09 diabetics patients were receiving glyburide, 09 used metformin, others were taking other associations as follows: glyburide and metformin (11 people), metformin and insulin (07), glyburide and insulin (01), insulin alone (05) and only 01 was not being treated with hypoglycemic agents due to be recently diagnosed. The drugs dosage was not changed during the study.

Each volunteer served as his own control. The study protocol followed three different stages and, each one, was carried out monitoring the patients fasting blood glucose levels. In the first stage, we summarize the fasting blood glucose values (FBS) of patients studied for the three months prior to the ingestion of the yellow passion fruit peel flour, when the patients were using drugs only. These data were obtained by analyzing the database of the UEPB Pharmaceutical Care Program (PROATENFAR) where patients have medical care. The second stage involved the measurement of all analysis parameters immediately before the intervention (T0) and during the use of supplemental feeding with the passion fruit flour for 30 and 60 days (T30 and T60). The third stage (GP) consisted of new tests after three months consumption of the flour in order to verify if blood glucose levels would return to baseline values or not.

Blood for laboratory tests was collected in the morning after a 12-hour fasting in the UEPB clinical analysis laboratory to obtain serum, which was packaged and transported in refrigerated containers to the Lauro Wanderley University Hospital Clinical Laboratory, where biochemical tests were realized. Four blood collections were carried out during the following stages: before supplementation (T0), during supplementation (T30 and T60) and after supplementation (GP), in the latter only fasting blood glucose was performed as a final control.

After the collection called T0 (before intervention), each patient under study received seven 30 g plastic bags of flour weekly, corresponding to 17.4 g of total fiber, being 6.3 g of soluble fiber and 11.1 g of insoluble fiber [18], to be eaten throughout the day along with food, which may include, among others, juices, fruits and milk. Because the flour is rich in pectin, a water-soluble fiber, patients were instructed to ingest at least two liters of water per day, since the low intake of this liquid could cause constipation [19].

### Anthropometric indicators

Among the anthropometric indicators were recorded: height, weight and Body Mass Index (BMI).

Height and weight were verified using a *Filizola* metal rod scale, measuring two meters and divided into 0.5 cm fractions with a 150 kg capacity and 100 g increase. The patient was positioned upright, looking towards the horizon, barefooted with their heels together, back straight and arms extended at their sides.

BMI was calculated by dividing weight (kg) by squared height (m^2^), using the BMI ranges, adopted for nutritional classification according to the World Health Organization [20].

### Laboratory data

Serum concentrations of glucose were determined using Biosystems^®^ kits and Biosystem^®^ A-25 analyzer; the determination of baseline insulin values was performed in the IMMULITE^®^ (immunometric procedure) to IR assess in patients and determination of the glycated hemoglobin (HbA1c) was determined by the Biosystems^®^ Turbidimetry method and reference values were based on those adopted by the International Federation of Clinical Chemistry (IFCC).

The insulin variables, HOMA IR and HOMA Beta, were performed in 32 patients of both genders of the same clinical group studied in the same period (19 females and 13 males). The losses were due to insufficient volume of the samples.

HOMA (Homeostasis Model Assessment), is a mathematical model that evaluates the relationship between glycemia and insulin and allows analyzing individuals in different IR severity conditions [21]. Furthermore, it is a low cost method, of easy implementation and large scale use [22]. Two indexes are extracted form it (HOMA IR and HOMA beta), designed to translate the insulin sensitivity and secretory capacity of beta cells. Thus, the model predicts insulinemia and glucose for a given sensitivity and insulin secretion capacity, assuming the RI would be the same in the liver and peripheral tissues in order to estimate insulin sensitivity throughout the body [23].

Reference values for the IR diagnosis - baseline insulin: 2-27 μUI/mL; HOMA IR up to 2.7 and HOMA Beta: 150-380 (“Pró –Sangue” Diagnosis Lab, where insulin dosages were made).

### Statistical analyses

At first the data were stored in a Microsoft Excel^®^ sheet. The descriptive statistical analysis was performed using the Epi-Info software, versions 6.04 and 3.4 and SPSS version 14, by applying the paired Student *t* test. For all statistical tests performed, we considered the 95% confidence interval and a significance level of 5% (p ≤ 0.05). Results were reported as Mean ± Standard Deviation (M ± SD).

## Results

The participants body weight remained constant in the first 30 days (p = 0.472) and was highest at 60 days (p = 0.000). In the nutritional diagnosis, BMI was used, which had at the beginning of treatment the overall average of 27.76 ± 3.24 kg/m2, at the end of the study, these values became 28.13 ± 3.16; 28.62 ± 2:48; 28.13 ± 3.16, respectively, showing that overweight was present both at baseline, and at the end of treatment (Table 1).

**Table 1:** Baseline values (weight, BMI) and average difference and standard deviations (SD) at 30 and 60 days of the study

**Parameters**	**Period**	**Average ± SD**	**Difference between the averages on the entire sample**		
			**T0/T30**	**T30/T60**	**T0/T60**
Weight(kg)	T0	66.78 ± 9.60	0.174 ± 1.57	-1.233 ± 1.48	-1.058 ± 1.72
	T30	66.60 ± 9.50	p = 0.472	p = 0.000	p = 0.000
	T60	67.84 ± 9.50			
BMI (kg/m^2^)	T0	27.76 ± 3.24	-0.140 ± 1.01	-0.279 ± 1.14	-0,372 ± 0,72
	T30	27.81 ± 3.41	p = 0.372	p = 0.116	p = 0.001
	T60	28.13 ± 3.16			

P ≤ 0.05 = Significance level; n = 43; *SD* = Standard deviation; *T0* = Baseline time; *T30* = 30 days; *T60* = 60 days; *BMI* = Body mass index.

A comparison between average values of fasting blood glucose of all patients (n = 43) performed in the period before and after the intervention, when patients did not use the passion fruit flour, showed that these values did not vary significantly (Table 2).

**Table 2:** Control of fasting blood glucose in the different study stages monitoring

**Glycemia (mg/dL)**	**Average ± SD**	**Difference between the averages**		
		**GA/G0**	**GA/GP**	**G0/GP**
GA	162.33 ± 69.83	p = 0.971	p = 0.797	p = 0.664
G0	162.56 ± 52.01			
GP	160.12 ± 56.02			

P ≤ 0.05 = significance level; n = 43; *SD* = standard deviation; *GA* = Blood glucose three months prior study; *GO* = Blood glucose at baseline time; *GP* = Blood glucose three months after supplementation.

A parallel of fasting blood glucose average levels of all patients (n = 43) immediately before (TO) and during the intervening period (T30 and T60) shows that there was a significant difference between them (Table 3). There was a 14.6% reduction in blood glucose during the first 30 days and 25.7% after 60 days when patients took the passion fruit flour as a daily food supplement. HbA1c also presented a significantly difference during the study, following the reduction in the average values of fasting blood glucose.

**Table 3:** Difference in the average baseline values and SD at 30 and 60 days of the patients glycemic profile

**Biochemical variables**	**Period**	**Average ± SD**	**Difference between the averages on the entire sample**		
			**T0/T30**	**T30/T60**	**T0/T60**
Glucose (≤ 110 mg/dL)	T0	162.55 ± 52.09	23.67 ± 32.45	18.05 ± 33.44	41.72 ± 38.11
	T30	138. 88 ± 41.46	p = 0.000	p = 0.001	p = 0.000
	T60	120.83 ± 36.72			
Glycosylated Hb (≤ 6.4%)	T0	6.58 ± 3.04	-	-	0.88 ± 2.62
	T60	5.71 ± 1.82			
	T30	185.07 ± 92.71			p = 0.032
	T60	161.21 ± 91.09			

P ≤ 0,05 = significance level; n = 43; *SD* = Standard Deviation; *T0* = baseline time; *T30* = 30 days; *T60* = 60 days.

As shown in Table 4, the insulin values remained constant even after supplementation while HOMA IR showed low levels with a significant difference at 60 days of the study. HOMA beta had higher average values in both the first four weeks of intervention and at the end of the study.

**Table 4:** Average value, SD, difference of baseline average values and at 30 and 60 days of insulin, HOMA RI and HOMA by gender

**Variable**	**Evaluation time**	**Average ± SD**			**Difference between averages by gender**					
					**T0/T30**		**T30/T60**		**T0/T60**	
		**Female**	**Male**	**p**	**Female**	**Male**	**Female**	**Male**	**Female**	**Male**
Insulin (μUI/mL)	T0	7.97 ± 3.18	4.52 ± 2.81	0.004	1.00 ± 2.88	-2.53 ± 2.73	-0.42 ± 3.24	1.31 ± 2.14	0.74 ± 3.83	-1.00 ± 1.47
	T30	6,86 ± 3.25	6.89 ± 4.87	0.923	p = 0.146	p = 0.006	p = 0.578	p = 0.047	p = 0.412	p = 0.031
	T60	7.20 ± 4.19	5.54 ± 4.06	0.662						
HOMA IR	T0	3.17 ± 1.65	1.76 ± 1.54	0.006	0.95 ± 1.31	-0.77 ± 1.17	0.21 ± 1.23	0.77 ± 1.09	1,21 ± 1,62	0.08 ± 0.49
	T30	2.28 ± 1.12	2,61 ± 2.46	0.684	p = 0.005	p = 0.035	p = 0.465	p = 0.026	p = 0.004	p = 0.584
	T60	2.04 ± 1.34	1.75 ± 1.65	0.907						
HOMA Beta	T0	36.84 ± 22.78	21.54 ± 12.23	0.037	-4.37 ± 19,69	-16.77 ± 16.70	-22.58 ± 40.21	-4.61 ± 25.47	-26.95 ± 41.07	-21.38 ± 19.59
	T30	41.21 ± 27.48	38.31 ± 23.57	0.611	p = 0.346	p = 0.003	p = 0.025	p = 0.526	p = 0.010	p = 0.001
	T60	63.79 ± 51.22	42.92 ± 27.92	0.259						

P ≤ 0.05 = significance level; n = 32; *SD* = Standard Deviation; *T0* = baseline time; *T30* = 30 days; *T60* = 60 days; *HOMA IR* = Homeostasis Model Assessment-insulin resistance; *HOMA Beta*.

As also shown in the table above there was no significant change in insulin values in females, whereas among men there was an increase in insulin levels after both the first 30 days and at the end of the study. Regarding HOMA IR checked at baseline, greater IR in females, which was decreased during the study; that did not occurred to males. For the HOMA Beta variable, an increase is seen in the average values in both groups throughout the study period, with a significant difference at 60 days of study for females. The males showed significance in their values in both established times.

## Discussion

Regarding the body weight no significant difference was observed during the study. One explanation for these data could be related to the short period of use of the passion fruit flour causing limitation on the population studied to assess the weight loss, gain or invariability.

Evaluated overweight, obese and placebo patients for 16 weeks suggesting that diet supplementation with soluble fiber for weight reduction could be beneficial because fiber induces satiety and a more favorable lipoprotein profile in the long run [24]. However, these results do not support the hypothesis that fiber supplements may have additional effects on weight loss, since they were not statistically significant in this group. Studied the effect of high dietary fiber intake (50 g) in patients with DM2, for 6 weeks, and the results were also not significant for body weight reduction [25].

Most individuals in this study were not able to keep their blood glucose levels in the range considered normal even using drug therapy. We analyzed the fasting blood glucose levels three months before the study and three months after the end of it and obtained, respectively, the average values of 162.33 mg/dl and 160.12 mg/dl; confirming that these patients actually were uncompensated and justifying the importance of using an alternative treatment method in an attempt to reduce blood glucose levels.

In the present study, we observed a statistically significant reduction in fasting plasma glucose at 30 and 60 days of treatment with the passion fruit flour. One possible explanation for this effect would be the presence of fibers in the food, mainly pectin, which form viscous mixtures (gel formation) which can change the gastric emptying time, increase satiety and delay the absorption of simple carbohydrates. Moreover, this gel is still able to form complexes with bile salts increasing the cholesterol excretion and may be used for cardiovascular disease, obesity, lipid disorders and diabetes mellitus type 2 treatment or prevention [10].

Besides the reduction in the average baseline glucose values, it was observed a significant reduction in HbA1c values between baseline and 60 days in patients treated with the passion fruit flour. These results are consistent with other studies, which demonstrated that fiber intake improves control and reduces the risk of chronic complications of type 2 diabetes [8,10,26,27]. However, in relation to the glycemic levels achieved, the results of different studies showed variations.

It is important to note that HbA1c has a fundamental role in monitoring the glycemic control in diabetic patients because it provides information about the retrospective index of plasma glucose (60 to 90 days of the exam). The great advantage of HbA1c consists in not suffering from significant fluctuations, as in the determination of plasma glucose, as well as being directly related to the risk of complications in patients with DMT2 [28]. In this study this dosage was performed only at 60 days of supplementation because it is the period of the clinical study.

Among patients with type 2 diabetes treated with glibenclamide and dietary restrictions that used conventional diet supplemented with *psyllium* (seed husk of *Plantago ovata*), we observed a decrease of 12.2% in glucose uptake (significant difference) and a not significant reduction in HbA1c [29]. Supplementing with passion fruit flour made in this study provided a 25.7% reduction in fasting blood glucose while in HbA1c it was 13.2%.

Other researchers have studied the effect of dietary fiber from rice bran involving diabetic patients treated with insulin. Hypoglycemic agents or diet control showed that the average fasting and postprandial blood glucose levels were reduced (p <0.001) when subjected to the diet with 40 g of the rice bran fiber during two weeks [30].

The effects of ingestion of food based on parameters recommended by the American Diabetes Association (ADA), containing moderate amounts of dietary fiber (total of 24 g, being 8 g of soluble fiber and 16 g of insoluble fiber) and also the consumption of higher fiber dietary levels (50 g in total, in equal proportions) in patients with type 2 diabetes, most of them in use of medication, within six weeks, were evaluated and compared by researchers who found a significant decrease (P = 0.04) in the average concentrations of plasma glucose and a slight reduction of HbA1c (P = 0.09) [25].

The consumption of the yellow passion fruit peel flour in patients with type 2 diabetes in the present study showed a significant increase in HOMA beta, both in general and in males and females during the established time, as there was a decrease in IR especially in females. These findings demonstrated that consumption of fiber is beneficial for the type 2 diabetes and CVD control. This corroborates with a study that evaluated fiber intake in diabetic patients in which was observed attenuation of IR [1].

The assay with hyperinsulinemic and hypertensive patients of both genders, who were fed with soluble fiber, indicated the improvement in insulin sensitivity [31]. Similarly, the findings of this study also showed an IR decrease, suggesting an increased sensitivity at 60 days of supplementation. Also significant beneficial effects of daily intake of soluble and insoluble fiber (25 g each) were observed for six weeks, in patients with type 2 diabetes under pharmacological treatment, which showed decrease in insulin levels [25]. However, insulin values were unchanged in this study population over the time established; with respect to the gender, there was a decrease in the figures only in males.

Some researchers studied 347 patients with metabolic syndrome diagnosed as normal, glucose intolerants and type 2 diabetes, and they found that the HOMA IR values increased while HOMA Beta diminished in patients with type 2 diabetes. This indicates therefore that the ability of beta cell secretion determines the occurrence of type 2 diabetes. Therefore, substances that act on insulin resistance decrease and increase the beta cell secretion function is relevant for the treatment of patients with the above-mentioned condition [25].

It was shown that dietary fiber improves the sensitivity to insulin in diabetics and non-diabetic [32,33] as well as in experimental animal models [34]. Research in spontaneously hypertensive rats predisposed to stroke suggested supplementation with *psyllium* soluble fiber effectively prevents IR by increasing the content of GLUT4 in the plasma membrane of skeletal muscle without activation of the PI3 kinase. One hypothesis for this increase may be related to a series of fatty acids that would stimulate the peroxisome proliferator activation receptorγ (PPARγ) whose activation increases the content of GLUT4 in adipocytes. It is unlikely that short chain fatty acids such as propionic and butyric acids, which results from the anaerobic bacteria fermentation of soluble dietary fiber in the colon, raise the GLUT4 muscle via PPARγ [35].

Although no mechanism has been described to explain the benefits of insoluble fiber, their use enhances insulin sensitivity in the body [36]. Ingestion of these fibers within the RDA [37] significantly accelerates insulin response, an effect that was associated with an increase in the postprandial active amounts of the glucose-dependent insulin tropic peptide incretin hormone (GIP), while the glucagon-like peptide 1 (GLP-1) was not affected [10]. In contrast, the soluble dietary fibers, such as oligofructose, have been described to increase the secretion and concentration of GIP and GLP-1 [38-40]. These short-term effects do not appear to be influenced by colonic fermentation. Thus, long-term studies, with the inclusion of individuals with damaged glucose metabolism should be performed in order to confirm these findings, as well as other techniques using animal models could be studied in order to elucidate the mechanisms involved in the effects of both soluble and insoluble fiber in insulin sensitivity and the role played by the incretin hormones in such mechanisms [10].

Another mechanism that may explain the effect of dietary fiber on insulin sensitivity is the attenuation of the glycemic response to carbohydrate intake through their mechanical action in the intestine, where they tend to slow the absorption of nutrients [40,41]. The reduction of serum glucose concentration decreases the amount of insulin necessary to capture it; over time, the reduction in insulin concentration in the environment can result in regulation of insulin receptors on the cell surface, thus increasing insulin sensitivity [34].

## Conclusions

Before the results obtained in this study, the intake of fiber-rich flour (pectin) suggests a favorable effect on insulin sensitivity during the eight-week period in the studied adults. These effects may reduce the risk of chronic complications of type 2 diabetes. Although larger and longer trials are needed to confirm these results and elucidate the mechanisms involved, evidence-based literature are enough to encourage increased consumption of foods rich in dietary fiber.

## Competing interests

The authors declare that they have no competing interests.

## Authors' contributions

All authors participated in the design, interpretation of the studies and analysis of the data; MSRQ and DIJ designed, supervised, conducted the study, interpreted data, performed the statistical analysis and wrote the manuscript. MALC carried out the biochemical determinations. JSM reviewed the writing of the manuscript. AUOSS provided the dietary supplement and helped with the project design. MFFMD and SCS edited the manuscript. All authors read and approved the final manuscript.
